# Block catiomers with flanking hydrolyzable tyrosinate groups enhance *in vivo* mRNA delivery *via* π–π stacking-assisted micellar assembly

**DOI:** 10.1080/14686996.2023.2170164

**Published:** 2023-03-16

**Authors:** Wenqian Yang, Takuya Miyazaki, Yasuhiro Nakagawa, Eger Boonstra, Keita Masuda, Yuki Nakashima, Pengwen Chen, Lucas Mixich, Kevin Barthelmes, Akira Matsumoto, Peng Mi, Satoshi Uchida, Horacio Cabral

**Affiliations:** a Department of Bioengineering, Graduate School of Engineering, The University of Tokyo, Tokyo, Japan; b Department of Radiology, Center for Medical Imaging, and State Key Laboratory of Biotherapy and Cancer Center, West China Hospital, Sichuan University, Chengdu, China; c Kanagawa Institute of Industrial Science and Technology, Ebina, Japan; d Department of Materials Science and Engineering, School of Materials and Chemical Technology, Tokyo Institute of Technology, Tokyo, Japan; e Institute of Biomaterials and Bioengineering, Tokyo Medical and Dental University, Tokyo, Japan; f Graduate School of Medicine, Kyoto Prefectural University of Medicine, Kyoto, Japan

**Keywords:** Messenger RNA, nanomedicine, polymeric micelles, π–π interaction, poly(ethylene glycol)-poly(glycerol), biocompatible

## Abstract

Messenger RNA (mRNA) therapeutics have recently demonstrated high clinical potential with the accelerated approval of SARS-CoV-2 vaccines. To fulfill the promise of unprecedented mRNA-based treatments, the development of safe and efficient carriers is still necessary to achieve effective delivery of mRNA. Herein, we prepared mRNA-loaded nanocarriers for enhanced *in vivo* delivery using biocompatible block copolymers having functional amino acid moieties for tunable interaction with mRNA. The block copolymers were based on flexible poly(ethylene glycol)-poly(glycerol) (PEG-PG) modified with glycine (Gly), leucine (Leu) or tyrosine (Tyr) *via* ester bonds to generate block catiomers. Moreover, the amino acids can be gradually detached from the block copolymers after ester bond hydrolyzation, avoiding cytotoxic effects. When mixed with mRNA, the block catiomers formed narrowly distributed polymeric micelles with high stability and enhanced delivery efficiency. Particularly, the micelles based on tyrosine-modified PEG-PG (PEG-PGTyr), which formed a polyion complex (PIC) and π–π stacking with mRNA, displayed excellent stability against polyanions and promoted mRNA integrity in serum. PEG-PGTyr-based micelles also increased the cellular uptake and the endosomal escape, promoting high protein expression both *in vitro* and *in vivo*. Furthermore, the PEG-PGTyr-based micelles significantly extended the half-life of the loaded mRNA after intravenous injection. Our results highlight the potential of PEG-PGTyr-based micelles as safe and effective carriers for mRNA, expediting the rational design of polymeric materials for enhanced mRNA delivery.

## GRAPHICAL ABSTRACT

**Figure:**
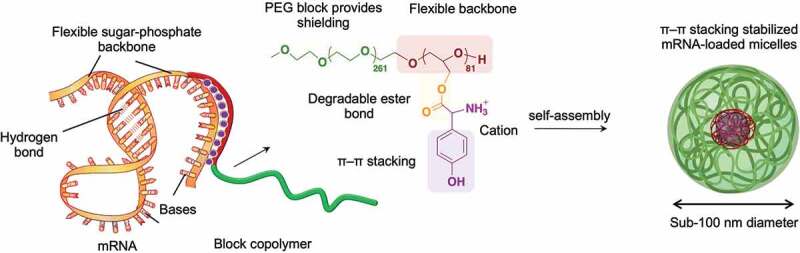

## 1. Introduction

Messenger RNA (mRNA) is at the center of many innovative genetic treatments due to its ability to generate a variety of therapeutic proteins in target cells [1]. This interest is driven by mRNA’s predictable expression profile, favorable safety characteristics, ability to express protein in many cell types (even non-dividing cells) [2], simple preparation, and flexible application [3]. Moreover, the recent approvals of SARS-CoV-2 vaccines confirmed the clinical applicability of mRNA [4,5]. However, in applications other than vaccines, the immunostimulatory properties of mRNA, its fragility in physiological environments and its inability to cross the cellular membrane have limited its implementation [3,6]. Thus, the development of safe and effective carriers is crucial to overcome the challenges faced by mRNA in further clinical applications [7].

Nanocarriers based on different materials, such as lipids, polymers and inorganic nanoparticles, are under intense investigation for mRNA delivery [7–11]. Among them, polyion complex (PIC) polymeric micelles using block catiomers with a hydrophilic neutral segment and a cationic block are attractive nanocarriers due to their protective core-shell nanostructure, accessible synthetic procedures and tunable features for enhancing mRNA delivery [3,12,13]. To promote the transport of mRNA to target cells *in vivo*, it is essential to design micelles capable of overcoming the enzymatic degradation of mRNA [14] and dissociation of the PIC by negatively charged glycans on cell surfaces and plasma [15,16]. Thus, enormous efforts have been dedicated to engineer polymeric structures for promoting both structural and mRNA stabilities [8,17–20]. In this regard, we have recently found that block catiomers containing flexible polycation segments based on polyether backbones stabilized the mRNA-loaded micelles against polyanion and enzymatic attack by enhancing the association with mRNA compared to catiomers having relatively more rigid polycation segments based on polyamide bonds [21]. Such strong polymer-nucleic acid interactions promoted the integrity in biological environments and improved cellular uptake [21,22], demonstrating the relevance of controlling polycation flexibility for improving delivery efficiency. Nevertheless, once the micelles reach the cytosol of the target cells, the strong interactions between the polymers and mRNA should be damped for facilitating mRNA release and boosting protein translation.

Herein, we developed mRNA-loaded micelles based on flexible block catiomers bearing hydrolyzable functional amino groups for improved structural stabilization in *in vivo* conditions and superior intracellular delivery. The functional groups were inspired by the great contribution of π-π stacking between RNA molecules and the aromatic residues in proteins to the stability of RNA-protein complexes [23,24]. Thus, we modified the side chain of flexible poly(ethylene glycol)-poly(glycerol) (PEG-PG) with tyrosine (Tyr) units for enhancing the structural integrity and protecting mRNA. The Tyr moieties were conjugated *via* hydrolyzable ester bonds for enabling mRNA release inside the cells, as well as reducing the cytotoxicity of the catiomer. To investigate the contribution of the π-π stacking on the delivery efficiency, we also prepared control block copolymers conjugated with glycine (Gly) and leucine (Leu), which would stabilize the micelle core through its hydrophobic isobutyl residue. Our results showed that the micelles prepared from the Tyr-modified PEG-PG (PEG-PGTyr) displayed excellent stability against polyanion dissociation and effectively protected mRNA in serum due to their PIC and π–π interactions. Moreover, the PEG-PGTyr-based micelles (PEG-PGTyr/m) significantly extended the half-life of the mRNA in the bloodstream after intravenous injection. In addition, PEG-PGTyr/m promoted the transfection *in vitro* and *in vivo* upon intramuscular injection, leading to significantly higher protein expression. These findings indicate an innovative approach for improving the stability and functionality of mRNA/polymer assemblies *via* π–π stacking to generate efficient delivery vehicles.

## 2. Materials and methods

### 2.1. Material

*n*-Butylamine and *β*-benzyl-L-aspartate-*N*-carboxyanhydride (BLA-NCA) were obtained from NOF Corporation (Tokyo, Japan). Ethylene oxide was obtained from 3M Japan Co., Ltd. (Tokyo, Japan). Tetrahydrofuran (THF) (super dehydrated, purity >99.5%) and Toluene (super dehydrated, purity >99.5+%) were purchased from Kanto Chemical, Co., Inc. (Tokyo, Japan). Ethanol, diethyl ether, *N,N*-dimethylformamide (DMF) and piperidine (purity >98.0%) were purchased from Fujifilm Wako Pure Chemical, Co., Inc. (Tokyo, Japan). 1,1,3,3-Tetramethylguanidine (purity >99.0%), 2-methoxyethanol (purity >99.0%) epichlorohydrin (purity >99.0%), triisobutylaluminum, *N,N*-dimethylformamide (purity >99.5%), 4-dimethylaminopyridine (purity >99.0%), 1-(3-dimethylaminopropyl)-3-ethylcordiimide hydrochloride (purity >98.0%), *N*-[(9*H*-fluoren-9-ylmethoxy)carbonyl]glycine (purity >98.0%), *N*-[(9*H*-fluoren-9-ylmethoxy)carbonyl]-L-leucine (purity >98.0%) and *N*-[(9*H*-fluoren-9-ylmethoxy)carbonyl]-L-tyrosine (purity >95.0%) were purchased from Tokyo Chemical Industry Co., Ltd. (Tokyo, Japan). Sodium hydroxide, hydrochloric acid and HEPES buffer were purchased from Sigma-Aldrich (St. Louis, MO, U.S.A). Fetal bovine serum (FBS), Dulbecco’s Modified Eagle’s Medium and Penicillin-Streptomycin were obtained from Thermo Fisher Scientific (Waltham, MA, U.S.A).

### 2.2. Cells

Human Embryonic Kidney cells 293 (HEK293) cells and human hepatocellular carcinoma HuH7 cells were obtained from Riken BioResource Center (Tsukuba, Japan).

### 2.3. Animals

All animal studies described below were approved by the Animal Care and Use Committee of the University of Tokyo (Tokyo, Japan) (KA21–1). Balb/c mice (7-week-old, female) were purchased from Charles River Laboratories (Yokohama, Japan).

### 2.4. Polymer synthesis

#### 2.4.1. Synthesis of α-methoxy-poly(ethylene glycol)-block-poly(glycerol) copolymer (PEG-PG) block copolymer

**Figure 1.**
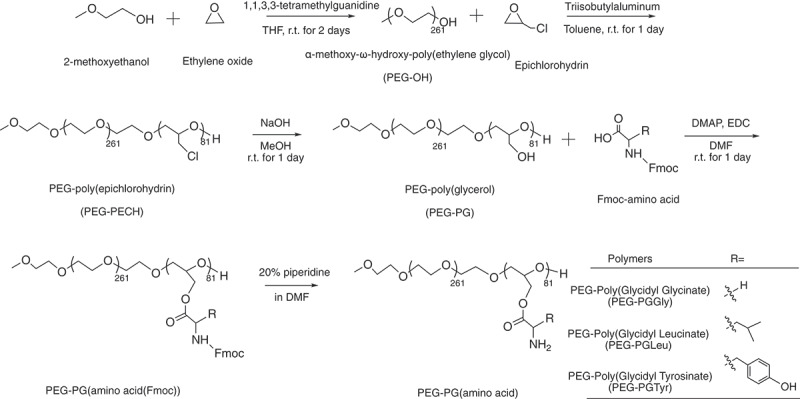
Scheme of polymer synthesis. Synthesis scheme of α-methoxy-poly(ethylene glycol)-block-poly(glycerol) (PEG-PG) copolymers from ring opening polymerization and amino-acid conjugated PEG-Poly(Glycidyl Glycinate) (PEG-PGGly), PEG-Poly(Glycidyl Leucinate) (PEG-PGLeu) and PEG-Poly(Glycidyl Tyrosinate) (PEG-PGTyr).

PEG-PLL was prepared as described in a previous paper [25]. The synthesis route of PEG-PG is shown in Figure 1. First, α-methoxy-ω-hydroxy-poly(ethylene glycol) (PEG-OH) was prepared through ring opening polymerization (ROP). Briefly, the 1,1,3,3-tetramethylguanidine (1.0 mmol, 130 µL) and 2-methoxyethanol (0.10 mmol, 7.9 µL) were dissolved in a solution of 60 mL anhydrous THF. The mixture was stirred for 10 min under argon atmosphere, and then liquid ethylene oxide (27 mmol, 1.4 mL, 0°C) was added to the solution through a cooled syringe. The reaction mixture was stirred at room temperature for 2 days under argon atmosphere and dried under reduced pressure to remove unreacted monomers and THF. Furthermore, epichlorohydrin (10 mmol, 784 µL) in 11 mL of anhydrous toluene was added to the polymer solution. The reaction mixture was stirred for 10 min under Argon atmosphere, followed by the addition of triisobutylaluminum (1.0 µmol, 2 mL) under Argon atmosphere. The reaction was left stirring at room temperature for 1 day. The reaction was then stopped by adding 3 mL of ethanol. The resulting polymer was precipitated in diethyl ether (400 mL) to obtain α-methoxy-poly(ethylene glycol)-block-poly(epichlorohydrin) (PEG-PECH) block copolymer. Then, elemental analysis was performed using a Vario MACRO cube Elemental Analyzer (Elemental, Germany) to determine the molecular weight of the polymer and the length of the PECH block. To prepare PEG-PG, 5 mL of 1 M NaOH (aq) was added to a stirred suspension of PEG-PECH (5.0 µmol,100 mg) in 5 mL methanol. After reacting at room temperature for 1 day, the polymer solution was purified by dialysis against 0.01 M HCl (aq) for 3 times and pure water for 3 times with a 6–8 kD MWCO dialysis membrane. Finally, PEG-PG was collected as a white powder after lyophilization. The polymer was characterized again by elemental analysis using a Vario MACRO cube Elemental Analyzer to confirm the removal of Cl.

#### 2.4.2. Synthesis of PEG-Poly (Glycidyl Glycinate) (PEG-PGGly), PEG-Poly(Glycidyl Leucinate) (PEG-PGLeu) and PEG-Poly(Glycidyl Tyrosinate) (PEG-PGTyr)

α-Methoxy-poly(ethylene glycol)-block-poly(glycidyl-amino acids) were synthesized by EDC hydrochloride mediated conjugations, as follows: PEG-PG (5.0 µmol,100 mg) was dissolved in 10 mL of DMF, followed by the addition of 4-dimethylaminopyridine (0.44 mmol, 54 mg) and 1-(3-dimethylaminopropyl)-3-ethylcordiimide hydrochloride (4.1 mmol, 637 mg). Then, 4.1 mmol of Fmoc-protected amino acids (1.2 g *N*-[(9*H*-fluoren-9-ylmethoxy)carbonyl]glycine; 1.4 g *N*-[(9*H*-fluoren-9-ylmethoxy)carbonyl]-L-leucine; 1.6 g *N*-[(9*H*-fluoren-9-ylmethoxy)carbonyl]-L-tyrosine) were added to the solution, and the resulting mixtures were stirred at room temperature for 1 day. The resulting polymer solutions were precipitated in diethyl ether (400 mL) to obtain PEG-PGGly(Fmoc), PEG-PGLeu(Fmoc) and PEG-PGTyr(Fmoc). Finally, the protecting Fmoc group was removed by treatment with a 20% solution of piperidine in DMF at a concentration of 10 mg/mL. The samples were precipitated in diethyl ether (400 mL) to obtain PEG-PGGly, PEG-PGLeu and PEG-PGTyr. The products were characterized by ^1^H-NMR using a JEOL EX400 spectrometer (Tokyo, Japan) at 400 MHz. The number- and weight-average molecular weight (*M*_*n*_, *M*_*w*_), and polydispersity index (PDI =*M*_*w*_*/M*_*n*_) of the polymer were tested by gel permeation chromatography using TOSOH HLC-8220 system (Tokyo, Japan) (eluent: 10 mm LiCl containing DMF; temperature: 40°C; flow rate: 0.5 mL/min; detector: refractive index). Polyethylene glycol standard ReadyCal calibration kit (Sigma-Aldrich, St. Louis, MO, U.S.A) with molar masses ranging from ~250 to ~45,000 Da was used for the calibration of the DMF-GPC system.

#### 2.4.3. Synthesis of Homo-poly(α,β-aspartic acid) (Homo-P(asp))

n-Butylamine and *β*-benzyl-L-aspartate-*N*-carboxyanhydride (BLA-NCA) were dissolved in super-dehydrated DMF under Argon flow. The mixture was then stirred at room temperature for 3 days. The resulting polymer solution was precipitated in diethyl ether to obtain homo-poly(*β*-benzyl-L-aspartate) (Homo-PBLA). Deprotection of the benzyl groups was performed by dissolving the prepared Homo-PBLA in a 0.5 N NaOH solution and stirring at room temperature for 1 h. The reaction solution was then dialyzed against pure water and lyophilized. The final product was characterized by ^1^H-NMR using a JEOL EX400 spectrometer (Tokyo, Japan) at 400 MHz (Supplementary Figure S1).

### 2.5. In vitro transcribed mRNA

*Gaussian luciferase* (Gluc) and *Firefly luciferase* (Fluc) mRNA were prepared from *in vitro* transcription. First, plasmid DNA temples were prepared by inserting the corresponding protein-coding sequences and 120 bp poly A/T sequence into the pSP73 vector (Promega, Madison, WI, U.S.A). Plasmid DNA was then linearized and transfected to produce Gluc and Fluc mRNA using mMESSAGE mMACHINE T7 Ultra Kit (Thermo Fisher Scientific, Waltham, MA, U.S.A). The resulting mRNA was purified with RNeasy Mini Kit (Qiagen, Hilden, Germany). mRNA concentration was finally determined by NanoDrop 3300 spectrophotometer (Thermo Fisher Scientific).

### 2.6. mRNA labelling

Gluc mRNA was labeled using *Label* IT Nucleic Acid Labeling Kit according to the manufacturer’s protocol. Briefly, 25 µg mRNA was mixed with 10× labeling buffer A and *Label* IT® regent at 1:2 (v:w) ratio of label reagent to mRNA. The resulting solution was incubated at 37°C for 90 min. Labeled mRNA was purified by using ethanol precipitation.

### 2.7. Hydrolysis of pendant moieties in PEG-PGGly, PEG-PGLeu and PEG-PGTyr

The detachment of the amino acids from PEG-PGGly, PEG-PGLeu and PEG-PGTyr was investigated by incubating 10 mg of the polymers in 10 mL pH 7.4 phosphate buffered saline (10 mM phosphate with 150 mm NaCl) and 10 mL pH 5.0 sodium acetate buffered saline (10 mM sodium acetate with 150 mM NaCl) at 37°C. At defined time intervals, the polymer solutions were placed in a Vivaspin 500 protein concentrator spin column (molecular weight cutoff (MWCO): 3,000 Da) and centrifuged at 5,000 rpm for 1 h to separate the detached amino acids from the polymers. Finally, the solution was lyophilized and the amount of amino acids was quantified by measuring the concentration of primary amines by fluorometric assay with fluorescamine.

### 2.8. Cytotoxicity

HEK293 cells were seeded on 96-well plates at 5 × 10^3^ cells/well. After 24 h, the cells were washed twice with PBS. Then, PEG-PGTyr, PEG-PGLeu, PEG-PGGly and PEG-PLL were added to cell culture media at concentrations of 0.02, 0.04, 0.08, 0.2, 0.4, 0.8, 1 mg/mL. After 24-h incubation, the cell viability was determined by cell-counting kit-8 (CCK-8; Dojindo Molecular Technologies Inc., Tokyo, Japan) assay following the manufacturer’s protocol.

### 2.9. Micelle preparation and characterization

mRNA and PEG-PGGly, PEG-PGLeu and PEG-PGTyr were dissolved in 10 mM HEPES buffer (pH 7.3) at 40 ng/µL and 1 mg/mL, respectively. The mRNA-loaded micelles were prepared *via* mixing freshly prepared polymer solution with mRNA solution at 1:1 volume. The polymer and mRNA amounts were fixed at the molar ratio of primary amines in polymer and phosphates in mRNA (N/P ratio) from 1 to 5. After mild vortexing, the resulting micelle solution was incubated at 4°C for 1 h to allow the micelles to stabilize. Then, electrophoretic analysis of micelles at varying N/P ratios ranging from 1 to 5 on 1% agarose gel (15 µL sample solution containing 500 ng of mRNA was applied to each well and mRNA was visualized using Midori Green Direct dye). The Z-average diameter and polydispersity index (PDI) of the micelles were characterized by dynamic light scattering (DLS) using a Zetasizer Nano ZS (Malvern Instruments Ltd., UK).

### 2.10. Micelle stability against polyanions

Micelles containing Cy5-labeled mRNA (20 ng/µL) were incubated with heparin at different S/P ([sulfate in heparin]/[phosphate in mRNA]) ratios. After 6-h incubation, fluorescence correlation spectroscopy (FCS) measurements were performed using an LSM-780 confocal laser scattering microscope (CLSM; Carl Zeiss AG, Oberkochen, Germany) with He-Ne laser (633 nm) scanning. The Cy5 dye (Lumiprobe Co., U.S.A) was used as a standard to calculate the diffusion coefficient of the Cy5-labeled mRNA and the micelles loading Cy5-labeled mRNA. The following theoretical model, which describes a general case of a sample containing M populations of fluorescent particles was used for fitting of autocorrelation curve. Each population of fluorescent particles was characterized by its diffusion time $\tau_{Di}$, $Q_{i}$ is the molecular brightness, $F_{i}$ is the fraction of the particle number and $\omega_{O}/\omega_{z}$ is the structure parameter [26].

$$G\left(\tau \right)=\frac{\sum_{i=1}^{M}{\left(Q_{i}\right)}^{2}F_{i}g_{i}\left(\tau \right)}{N{\left(\sum_{i=1}^{M}Q_{i}F_{i}\right)}^{2}}$$

(1) $$g_{i}\left(\tau \right)=\frac{1}{1+\left(\tau /\tau_{Di}\right)}\sqrt{\frac{1}{1+\left(\tau /\tau_{Di}\right){\left(\omega_{O}/\omega_{z}\right)}^{2}}}$$

### 2.11. Hydrolysis of esters and esterase-responsive mRNA release

Micelles (mRNA concentration: 50 ng/μL) containing Cy5-labeled GLuc mRNA were incubated in HEPES buffer (pH 7.4) with and without esterase. The final esterase concentration was 2.85 µg/mL. After 24-h incubation, the micelles were incubated with heparin at different S/P ([sulfate in heparin]/[phosphate in mRNA]) ratios, the final mRNA concentration was 20 ng/µL. After 1-h incubation, fluorescence correlation spectroscopy (FCS) measurements were performed as described in *2.10*.

### 2.12. Micelle stability in serum

Micelles containing GLuc mRNA were incubated with 50% fetal bovine serum (FBS; Thermo Scientific Fisher Inc., U.S.A) at 37°C for 15 min. The final mRNA concentration was 50 ng/μL. The mRNA was then extracted by the RNeasy Mini Preparation Kit (Qiagen, Hilden, Germany). The mRNA was then reverse-transcribed with the ReverTra Ace qPCR RT Master Mix kit (Toyobo Life Science, Osaka, Japan), followed by quantitative real-time PCR (qRT-PCR) using an ABI Prism 7500 Detector (Applied Biosystems, Foster City, CA, U.S.A) and a primer pair for GLuc (Forward: TGAGATTCCTGGGTTCAAGG, Reverse: GTCAGAACACTGCACGTTGG).

### 2.13. π–π stacking assessment by Tyr fluorescence quenching

A solution of PEG-PGTyr (200 μL; 1 mg/mL) was placed in 96-well plates. Next, mRNA (40 ng/µL) and Homo-PAsp solution (1 mg/mL) was added to the polymer at N/P = 3 and N/[COO] = 3, respectively. The fluorescence intensity of Tyr in the solution was then evaluated at the excitation wavelength (λ_ex_) of 280 nm and the emission wavelength (λ_em_) of 350 nm by multimode microplate reader (Tecan Group Ltd., Switzerland).

### 2.14. Cellular uptake and endosome escape study

HuH-7 cells were seeded on an eight-well chambered borosilicate cover glass (Lab Tek) a day before transfection at a density of 10,000 cells/well and incubated for 24 h in DMEM containing 10% FBS and 1% penicillin/streptomycin under 5% CO_2_ at 37°C. Next, naked Cy5-labeled mRNA and Cy5-labeled mRNA-loaded micelles were applied to each well (700 ng mRNA per well, relative fluorescence intensity: 400 RFU). The cellular uptake of the micelles was evaluated after 8 h using an LSM-780 with a 40 × objective (C-Apochromat, Carl Zeiss, Germany). The lysosomes were stained by LysoTracker Green, and the nuclei were stained by Hoechst 33,342. To evaluate the intracellular distribution of mRNA loaded in micelles, the rate of colocalization of the Cy5 signal with that of LysoTracker Green was quantified.

### 2.15. In vitro transfection

HuH-7 cells were seeded in a 96-well plate a day before transfection at a density of 30,000 cells/well. After 24-h incubation in DMEM containing 10% FBS and 1% penicillin/streptomycin under 5% CO_2_ at 37°C, the cell culture medium was replenished with media containing GLuc mRNA, PEG-PGGly/m and PEG-PGTyr/m containing 1000 ng of Gluc mRNA. After 24 h, the luminescence intensities of 50 μL supernatant were evaluated by GloMax 96 Microplate Luminometer (Promega, Madison, WI, U.S.A) using Renilla Luciferase Assay System (Promega, Madison, WI, U.S.A).

### 2.16. Blood circulation

Micelle solutions (200 μL) containing 4 μg of mRNA were administered to BALB/c mice (female, 7 weeks old, Charles River Laboratories Japan Inc., Kanagawa, Japan) by intravenous injection. Blood (2 μL) was collected from the tail vein of each mouse after 2.5, 5 and 10 min of administration, and the isolation of mRNA from the blood was performed by immediately mixing the blood samples with 350 μL 1% 2-mercaptoethanol containing RLT buffer from the RNeasy Mini Kit (Qiagen, Hilden, Germany). mRNA was then purified using RNeasy Mini Kit (Qiagen, Hilden, Germany), followed by reverse transcription of mRNA using ReverTra Ace qPCR RT Master Mix kit (Toyobo Life Science, Osaka, Japan). Then, qRT-PCR analysis was performed as described in point *2.12*.

### 2.17. In vivo transfection

Micelle solutions (70 μL) containing 5 μg of firefly luciferase mRNA (Fluc) were administered by intramuscular injection to Balb/c mice (female, 7 weeks-old; Charles River Laboratories Japan, Inc). After 9 h of administration, the mice were intraperitoneally injected with 200 μL 50 mg/mL luciferin solution. The mice were anesthetized with isoflurane *via* inhalation in a box chamber, and the luciferase expression was evaluated after 15 min using an *in* *vivo* bioluminescence imaging system (IVIS Spectrum SP-BFM-T1, PerkinElmer, Waltham, MA, U.S.A).

## 3. Results

### 3.1. Block copolymer synthesis

α-Methoxy-ω-hydroxy-poly(ethylene glycol) (PEG-OH) was synthesized *via* ROP initiated by the terminal hydroxyl of 2-methoxyethanol, with an organometallic catalyst (triisobutylaluminum). The synthesized PEG-OH showed a unimodal molecular weight distribution (Supplementary Figure S3a) with 261 units as determined by ^1^H-NMR (Supplementary Figure S2). Next, the PEG-OH was used as the initiator for the ROP of epichlorohydrin. Triisobutylaluminum was again used as a catalyst. The resulting PEG-PECH polymer showed a unimodal molecular weight distribution (Suplementary Figure S3b). Elemental analysis of the chloride atoms in the PECH segments determined the number of PECH units in the block copolymer to be 81 (Supplementary Table S1). After treating the polymer with NaOH, the chloride groups on the side chain were completely converted to hydroxyl groups, resulting in PEG-PG as confirmed by the disappearance of the Cl atoms in the elemental analysis. Unimodal molecular weight distribution is also observed for PEG-PG (Suplementary Figure S3c). The hydroxyl groups of the PEG-PG were conjugated with three different Fmoc-protected amino acids (Gly-Fmoc, Leu-Fmoc and Tyr-Fmoc) *via* condensation reaction to form hydrolyzable ester bonds. Thus, the amino acid-modified block copolymers were obtained by deprotecting the Fmoc groups of the amino acids, resulting in PEG- PEG-PGGly, PEG-PGLeu and PEG-PGTyr. The conjugation rates of glycine, leucine and tyrosine to PEG-PG were calculated to be 92.5%, 98.7% and 93.7% by ^1^H-NMR, respectively (Table 1 and Supplementary Figure S4). The block copolymers also maintained narrow molecular weight distributions (*M*_*w*_/*M*_*n*_), ranging from 1.03 to 1.08 (Table 1).

**Table 1.** Characterization of PEG-PGGly, PEG-PGLeu and PEG-PGTyr.

Polymers	*M*_*w*_/*M*_*n*_^a^	DP of PEG^b^	DP of PG^c^	Amino acid conjugation rate (%)^d^
PEG-PGGly	1.05	261	81	92.5
PEG-PGLeu	1.03	261	81	98.7
PEG-PGTyr	1.08	261	81	93.7

^a^Measured by GPC in DMF. ^b,d^Determined by 1H-NMR. ^c^Determined by elemental analysis.

### 3.2. Detachment of amino acids and cytotoxicity of polymers

**Figure 2.**
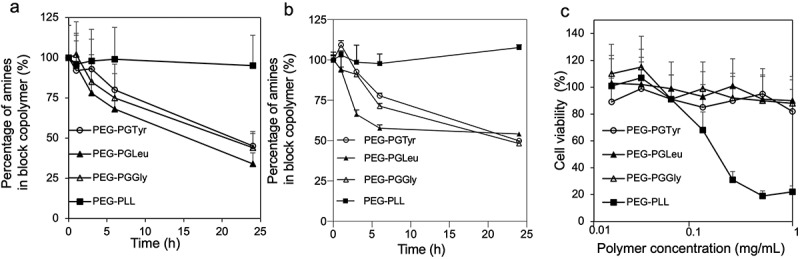
Evaluation of amino acid detachment and cytotoxicity of polymers. Remaining amines in the block copolymers after incubation in pH 7.4 10 mM PBS (a) and pH 5.0 10 mM sodium acetate (b) at 37°C determined by fluorometric assay with fluorescamine. Data are presented as the average ±  S.D. (n = 3). (c) Cytotoxicity of polymers against HEK293 cells. The cells were incubated with the polymers for 24 h. Data are presented as the average ± standard deviation (n = 3).

The hydrolytic degradation of the PEG-PGGly, PEG-PGLeu and PEG-PGTyr was evaluated by dissolving the polymers at pH 7.4 10 mM PBS and pH 5.0 10 mM sodium acetate at 37°C. The degradation was monitored by measuring the amount of free amino acid in the solution as a function of exposure time. Comparable detachment of amino acids from the polymers was observed at conditions mimicking extracellular pH (pH 7.4) and endosomal pH (pH 5.0) (Figure 2(a,b)). Thus, after 6-h incubation, more than 25% of the conjugated amino acids were detached, while at 24 h around 50% of the amines were detached. The control PEG-PLL remained stable throughout the experiment Figure 2(a,b). The detachment of the amino acids would be beneficial for releasing the mRNA after cellular uptake, as well as reducing the cytotoxicity by decreasing the positive charges in the polycation segment.

In this regard, the cytotoxicity of the block copolymers was evaluated in HEK293 cells after 24-h incubation. The results showed that the cell viability was not affected by PEG- PEG-PGGly, PEG-PGLeu and PEG-PGTyr (Figure 2(c)). In contrast, the addition of conventional PEG-PLL to the cells resulted in a much lower cell viability. These results support the enhanced safety of PEG-PGGly, PEG-PGLeu and PEG-PGTyr block copolymers.

### 3.3. Micelle formation and characterization

PIC micelles were assembled after mixing PEG-PGGly, PEG-PGLeu and PEG-PGTyr, with mRNA in 10 mM HEPES buffer (pH 7.3) at different N/P ratios. The condensation of the mRNA by polymers at various N/P ratios was analyzed by gel retardation assay and DLS measurement. During electrophoresis, the micelles, which are less negatively charged and heavier than free mRNA, are retained in the wells. In contrast, uncomplexed mRNA can migrate into the gel. Supplementary Figure S5 shows that the N/P ratio of 3 was sufficient to condense the mRNA in PEG-PGGly/m. Also, the PDI of the PEG-PGGly/m decreased as the N/P increased from 1 to 3 (Supplementary Figure S6b). The complexation was more efficient with PEG-PGLeu/m and PEG-PGTyr/m. mRNA was complexed and maintained in the wells even at N/P ≥2 (Supplementary Figure S5). For better comparison, the derived count rate of micelles at N/P 2-5 are normalized to N/P 1. At N/P = 3, micelles with a z-averaged diameter of around 60 nm, and PDI below 0.2 were obtained for all the polymers (Supplementary Figure S6, Table 2). At N/P equal to and higher than 3, the normalized the derived count rate of all the micelles reached a plateau, supporting the formation of the micelles (Supplementary Figure S6). These results are in agreement with our previous reports on mRNA-loaded PIC micelles [21,22].

**Table 2.** Size of micelles determined by DLS.

Samples	N/P	Z-average diameter (nm)	Polydispersity Index (PDI)
PEG-PGGly/m	3	58	0.19
PEG-PGLeu/m	3	60	0.15
PEG-PGTyr/m	3	55	0.19

### 3.4. Micelle stability and esterase-responsive mRNA release

**Figure 3.**
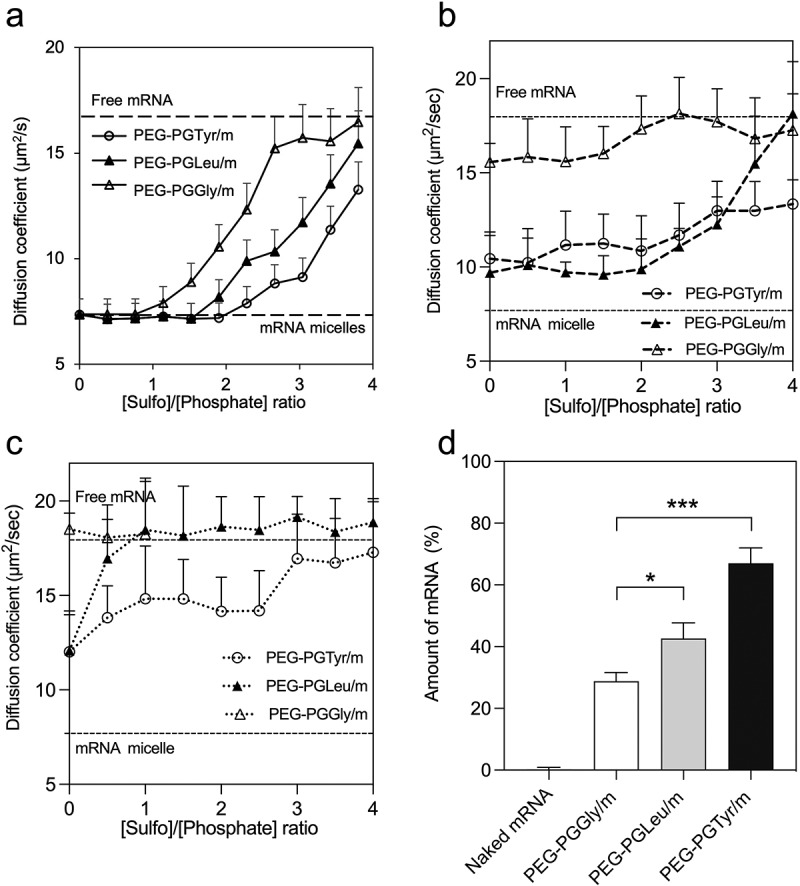
Stability and release of mRNA-loaded micelles. (a) Diffusion coefficient of fresh micelles after incubation with heparin at different S/P (sulfate in heparin/phosphate in mRNA) ratios for 6 h. (b) After 24-h incubation in HEPES buffer (pH 7.4), the micelles were incubated with heparin at different S/P (sulfate in heparin/phosphate in mRNA) ratios for 1 h, diffusion coefficient was recorded. (c) After 24-h incubation with esterase, micelles were then incubated with heparin at different S/P (sulfate in heparin/phosphate in mRNA) ratios for 1 h, diffusion coefficient was recorded. The upper and lower dashed lines represent the diffusion coefficients of the free mRNA and mRNA loaded micelles, respectively. Data are presented as the mean ± S.D. (n = 10). (d) Remaining mRNA percentage after incubating mRNA-loaded micelles with 50% FBS at 37°C for 15 min, followed by reverse transcription and qRT-PCR quantification. Data are presented as the mean ± S.D. (n = 3). Statistical significance was calculated by one-way ANOVA test. The difference was considered statistically significant with *p < 0.05, ***p < 0.001.

PIC-based carriers are susceptible to be disassociated by polyion exchange when exposed to anionic macromolecules (*i.e.* glycosaminoglycans on cell surfaces) [16,27], which can lead to the premature release and degradation of mRNA in the extracellular space. Thus, we incubated our mRNA-loaded micelles with negatively charged heparin at different [sulfate in heparin]/[phosphate in mRNA] (S/P) ratios for 6 h to compare their stability against polyanions. In this experiment, we used Cy5-labeled mRNA to trace the diffusion coefficient by FCS [28]. Thus, when Cy5-labeled mRNA is loaded into the micelles, the diffusion coefficient decreases (Figure 3(a)). However, when the mRNA is released, the diffusion coefficient increases. For PEG-PGTyr/m, the diffusion coefficient remained stable at S/P = 2, indicating that the micelles maintained their integrity even when the amount of negative charges in the polyanion doubles that in mRNA. On the other hand, the diffusion coefficient of PEG-PGGly/m started to increase at S/P = 1. Moreover, at S/P = 2, around 50% of the mRNA in PEG-PGGly/m has been released. These results indicate that PEG-PGGly/m is less stable than PEG-PGTyr/m. The introduction of the hydrophobic isobutyl groups in PEG-PGLeu/m improved the stability, but to a lower extent when compared to PEG-PGTyr/m (Figure 3(a)). These results suggest the importance of the aromatic residue in Tyr for stabilizing the micelles against polyanions.

We have characterized the polyanion stability of the micelles after incubating them for 24 h in HEPES buffer (pH 7.4) with and without esterase. After 24-h incubation in HEPES, the diffusion coefficients of micelles were increased even at sulfate-to-phosphate ratio (S/P) = 0 (Figure 3(b)), suggesting that the molecular weight of the micelles has been decreased. All the micelles were dissociated at lower concentration of polyanions, which was expressed as the S/P in heparin and mRNA, respectively (Figure 3(b)). When the micelles were exposed to esterase, the micelle dissociation occurred at lower S/P (Figure 3(c)). The results showed that PEG-PGGly/m incubated with esterase was already dissociated even at S/P = 0. PEG-PGLeu/m and PEG-PGTyr/m incubated with esterase were more stable than PEG-PGGly/m incubated with esterase, though they dissociated at lower S/P than the micelles without esterase. These data confirm the importance of the amino acids for stabilizing the micelles, and indicate that the side groups of the polymers can be hydrolysed to accelerate the release of mRNA. Also, the micelles exhibited esterase-promoted mRNA release, which could have considerable advantages for intracellular activation in some cells, for example, cancer cells [29–31].

### 3.5. mRNA protection ability against serum

mRNA therapeutics should also be protected against enzymatic attack following *in vivo* administration. To estimate the ability of the micelles to protect the mRNA cargo, we further incubated the micelle in 50% FBS, which simulates the harsh *in vivo* conditions [32]. The integrity of mRNA was then determined by quantitative real-time PCR (qRT-PCR). After 15 min incubation, around 70% of the mRNA was still detectable in PEG-PGTyr/m. In contrast, approximately 40% and 30% of the mRNA were detectable in PEG-PGLeu/m and PEG-PGGly/m, respectively (Figure 3(d)). The stability enhancement was more pronounced in PEG-PGTyr/m compared to PEG-PGLeu/m, which is stabilized by the hydrophobicity of the isobutyl groups. These results suggest that PEG-PGTyr achieved a remarkable stabilization effect, which is probably related to the ability of the polymer to form π–π stacking with mRNA.

### 3.6. π–π stacking assessment by Tyr fluorescence quenching

**Figure 4.**
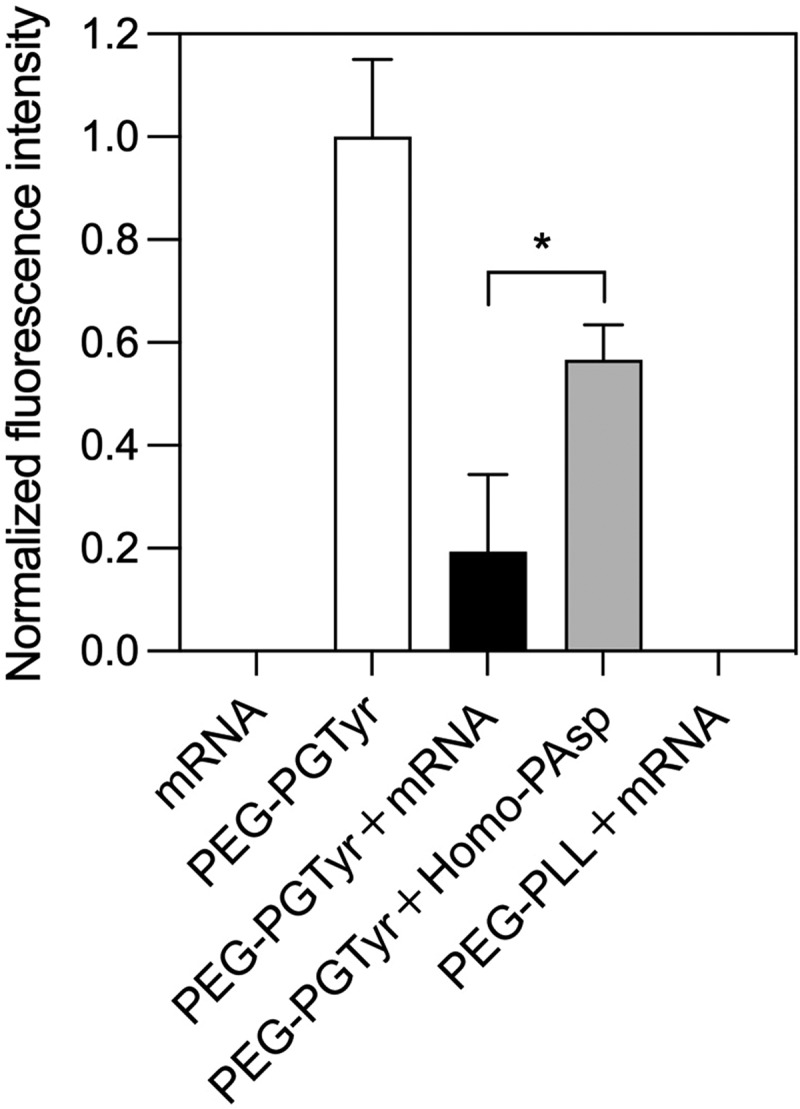
π–π stacking assessment by Tyr fluorescence quenching. Normalized fluorescence intensity of PEG-PGTyr polymer and corresponding fluorescence intensities of the mRNA, polymer and micelles. Data are presented as the mean ± S.D. (n = 3). Statistical significance was conducted using a two-tailed Student’s t-test. The difference was considered statistically significant with *p < 0.05.

In order to determine the π–π stacking between PEG-PTyr and mRNA in the micelles, we investigated the fluorescence quenching of Tyr moieties in the polymer. Tyr groups have intrinsic fluorescence (λex ~280 nm, λem ~350 nm), and π–π stacking can quench the fluorescence by electron transfer [33,34]. Thus, we investigated the change of fluorescence signal from the Tyr groups of PEG-PGTyr polymers after forming micelles with mRNA. For comparison, we also assembled micelles of PEG-PGTyr polymers with anionic Homo-PAsp, which does not have aromatic residues. We also checked the fluorescence signal of mRNA alone and PEG-PLL/m loading mRNA as controls for the conducted fluorescence emission measurements. The results showed that no fluorescence was detected in these controls (Figure 4). There is a strong decrease in Tyr fluorescence intensity in PEG-PGTyr/m loading mRNA. The fluorescence quenching of PEG-PGTyr/m was significantly higher than that of the micelles loading Homo-PAsp (Figure 4). These results support the π–π stacking between the aromatic Tyr groups and mRNA.

### 3.7. In vitro activity

**Figure 5.**
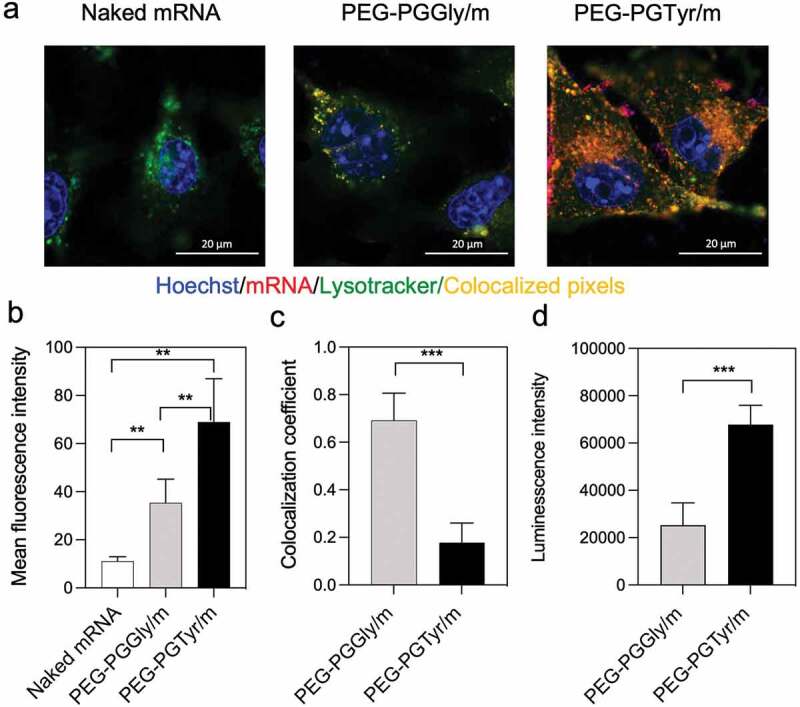
*In vitro* delivery of mRNA-loaded micelles. (a) Representative CLSM images of HuH7 cells incubated with micelles for 8 h (scale bar = 20 μm; Blue: Hoechst; Red: Cy5-labeled mRNA; Green: Lysotracker; yellow: colocalization of mRNA and Lysotracker). (b) Cellular uptake quantified by the mean fluorescence intensity of Red channel from 20 cells in each sample. Data are presented as the mean ± S.D. (n = 20). (c) Colocalization coefficients of Red-Green channels quantified from 10 cells in each sample. Data are presented as the mean ± S.D. (n = 10). (d) Transfection in HuH7 cells after 24 h treatment with micelles encapsulating GLuc mRNA. Data are presented as the mean ± S.D. (n = 4). Statistical significance was conducted using one-way ANOVA and Tukey’s post hoc tests (b) and two-tailed Student’s t-test (c,d). The difference was considered statistically significant with **p < 0.01, ***p < 0.001, denoted with asterisks.

We then explored the *in vitro* performance of the mRNA-loaded PIC micelles. The ability of the micelles for intracellularly delivering mRNA was studied in Huh7 cells. The uptake was visualized by CLSM using micelles loading Cy5-labeled mRNA. Thus, by quantifying the Cy5 fluorescence intensity, we found that micelles improved mRNA uptake *in vitro* remarkably (Figure 5). Moreover, the cellular uptake of PEG-PGTyr/m was about twofold higher than that of PEG-PGGly/m (Figure 5(b)). The cellular uptake enhancement of PEG-PGTyr/m could be associated with their high stability against polyanion exchange, which may allow them to effectively overcome the glycan barrier on the cell surface.

The ability of the micelles to escape from the endosomes was then evaluated by studying the endosomal colocalization. The endo/lysosomes were labeled with LysoTracker Green, and the mRNA in the endosomes was visualized as yellow pixels in the CLSM images (Figure 5(a)). The colocalization rate of mRNA and endo/lysosomes was quantified by the colocalization coefficient. A low colocalization coefficient indicates a high endosome escape ability. After 8-h incubation of the micelles with the HuH7 cells, PEG-PGTyr/m demonstrated a strong endosomal escape with decreased colocalization coefficient (Figure 5(c)), which was significantly lower than that of PEG-PGGly/m. The mechanism of the enhanced endosomal escape of PEG-PGTyr/m may be related to the hydrophobic Tyr residue, which may facilitate the destabilization of the endosomal membrane destabilization, as recently observed for carriers bearing aromatic amino acids [35–37].

The transfection ability of the micelles was studied in Huh7 cells after incubation with GLuc mRNA-loaded micelles. Herein, we used a naturally secreted Gluc as a reporter to assess gene expression in culture cells. The protein translation ability was determined by assessing the bioluminescence signal. PEG-PGTyr/m resulted in higher transfection efficiency compared to the PEG-PGGly/m (Figure 5(d)). We believe that this result can be associated with the enhanced stability against serum by π–π interaction and the improved endosomal escape of the PEG-PGTyr/m.

### 3.8. mRNA delivery performance in vivo

**Figure 6.**
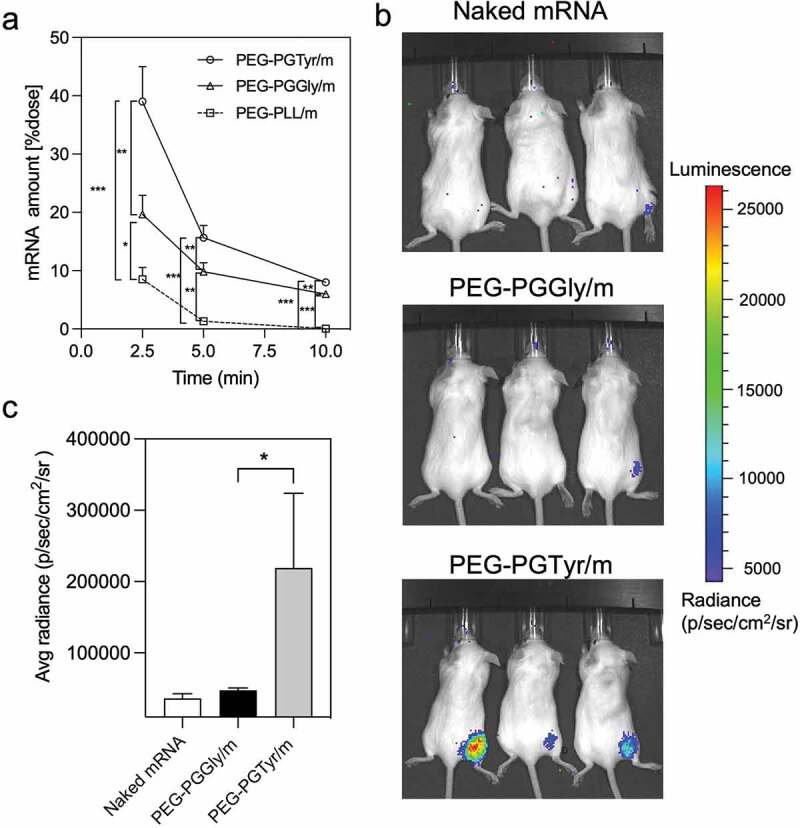
*In vivo* performance of mRNA-encapsulating micelles. (a) Remaining mRNA amount in the bloodstream of mice after intravenous injection of mRNA-loaded micelles containing 4 μg of mRNA. Data were shown as means ± S.D. (*n* = 5). Statistical significance was conducted using one-way ANOVA and Tukey’s post hoc tests. (b) Representative *in vivo* bioluminescence images 9 h after intramuscular injection of Fluc mRNA, PEG-PGGly/m and PEG-PGTyr/m (5 μg mRNA per mouse, n = 3). (c) Quantification of the luminescence signals from the images in b. Data were shown as means ± S.D. (n = 3). Statistical significance was conducted using a two-tailed Student’s t-test. The difference was considered statistically significant with *p < 0.05, **p < 0.01, ***p < 0.001, denoted with asterisks.

The ability of the micelles to deliver mRNA was further tested in mice. We first studied the capacity of the micelles to extend the circulation in the bloodstream, which is the ultimate experiment to determine the stability improvement of the micelles. Thus, a qRT-PCR-based assay was applied to assess the mRNA integrity in blood after intravenously injecting the micelles. The results showed that PEG-PGTyr/m prolonged the circulation of mRNA in the blood, with almost 40% of the injected dose remaining at 2.5 min after injection (Figure 6(a)). This value is twofold higher than that of the PEG-PGGly/m, indicating the superior performance of the PEG-PGTyr/m during blood circulation. Moreover, our previous report showed that mRNA-loaded polymeric micelles based on flexible catiomers achieved around 25% of the injected dose at 2.5 min, which is in agreement with the PEG-PGGly/m [21]. Also, PEG-PLL/m resulted in a significantly lower mRNA amount than the PEG-PGGly/m and PEG-PGTyr/m, with 8.5% of the injected dose remaining at 2.5 min after injection (Figure 6(a)). Free mRNA cannot be detected in blood 2.5 min after injection due to its fast degradation [32]. At longer time points, the mRNA of PEG-PGTyr/m was still detectable, attaining almost 10% of the injected dose at 10 min after injection. These findings confirmed the high stability of PEG-PGTyr/m in biological settings, and could be attributed to the interactions of the tyrosyl groups and mRNA’s bases in the core of the micelles.

The *in vivo* mRNA transfection was evaluated in mice after intramuscular administration of Fluc mRNA-loaded PIC micelles. Here, we used Fluc as a reporter to study gene expression in mice due to its non-secreted nature, which makes it beneficial to study the position of protein expression. Nine-hours after administration, the bioluminescent signals were seen at the injected site (Figure 6(b)). PEG-PGTyr/m treated mice showed strong bioluminescent signals, while almost no bioluminescence was detected from PEG-PGGly/m and naked mRNA (Figure 6(b,c)). These results clearly support the ability of PEG-PGTyr/m to improve the efficacy of mRNA delivery *in vivo* after intramuscular injection, suggesting the potential of this system for vaccination use.

## 4. Conclusion

Our study showed the enhanced mRNA delivery efficiency of polymeric micelles based on flexible block catiomers modified with functional amino acid moieties. We demonstrated that this system has minimal cytotoxicity due to the inclusion of hydrolyzable ester bonds between the amino groups and the polycation backbone. Moreover, the polymers having pendant tyrosyl moieties stabilized the micelle structure and effectively protected mRNA in the core by promoting π–π stacking with mRNA, which resulted in improved *in vitro* and *in vivo* delivery efficiency. These findings provide an innovative strategy for improving the stability and functionality of mRNA/polymer assemblies with high efficacy, and promote the rational design of polymeric materials for enhanced mRNA delivery.

## Supplementary Material

Supplemental Material
