# Supplementary material for: Block catiomers with flanking hydrolyzable tyrosinate groups enhance in vivo mRNA delivery via π–π stacking-assisted micellar assembly
Source: Sci Technol Adv Mater. 2023 Mar 16;24(1):2170164. doi: 10.1080/14686996.2023.2170164 (PMC10026751; doi:10.1080/14686996.2023.2170164)
Supplement: Supplemental Material [file TSTA_A_2170164_SM6755.pdf]

Supplementary Information

Block catiomers with flanking hydrolyzable tyrosinate groups enhance *in vivo* mRNA delivery *via* π–π stacking-assisted micellar assembly

Wenqian Yang[a,b#], Takuya Miyazaki[c#], Yasuhiro Nakagawa[d], Eger Boonstra[a], Keita Masuda[a], Yuki Nakashima[a], Pengwen Chen[a], Lucas Mixich[a], Kevin Barthelmes[c], Akira Matsumoto[e], Peng Mi[b], Satoshi Uchida[f], Horacio Cabral[a*]

[a]Department of Bioengineering, Graduate School of Engineering, The University of Tokyo, Tokyo, Japan, [b]Department of Radiology, Center for Medical Imaging, and State Key Laboratory of Biotherapy and Cancer Center, West China Hospital, Sichuan University, Chengdu, China, [c]Kanagawa Institute of Industrial Science and Technology, Ebina, Kanagawa, Japan, [d]Department of Materials Science and Engineering, School of Materials and Chemical Technology, Tokyo Institute of Technology, Tokyo, Japan, [e]Institute of Biomaterials and Bioengineering, Tokyo Medical and Dental University, Tokyo, Japan, [f]Graduate School of Medicine, Kyoto Prefectural University of Medicine, Kyoto, Japan.

#These authors contributed equally to this work.

Corresponding author:
Horacio Cabral
E-mail address: horacio@bmw.t.u-tokyo.ac.jp

Department of Bioengineering, Graduate School of Engineering, The University of Tokyo, 7-3-1 Hongo, Bunkyo-ku, Tokyo 113-8656, Japan

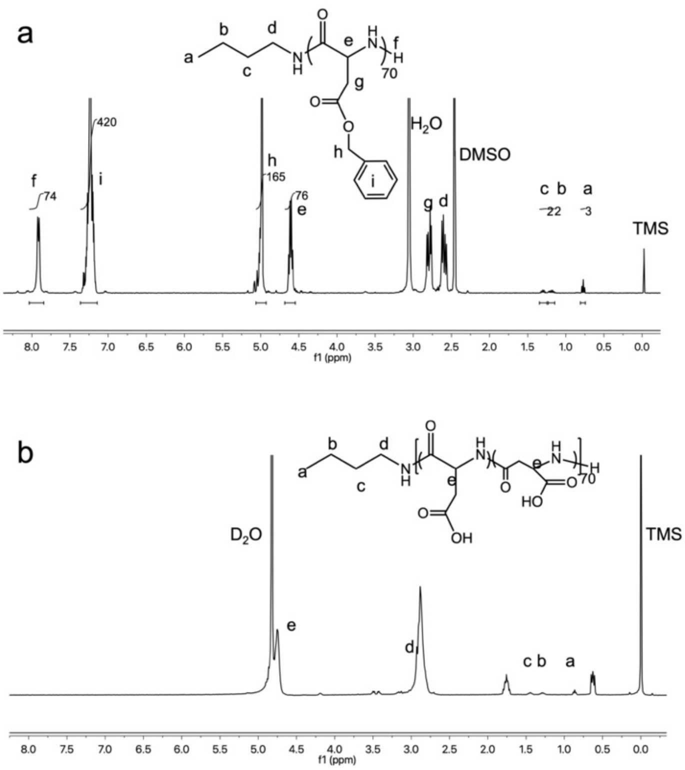

Supplementary Figure 1. ^{1}H-NMR of a) Homo-PBLA (polymer concentration: 10 mg/mL, solvent: DMSO, and temperature: 80 °C), b) Homo-PAsp (polymer concentration: 10 mg/mL, solvent: D_2O, and temperature: 25 °C)

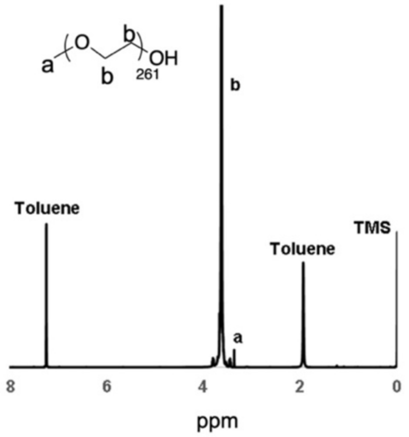

Supplementary Figure 2. ^{1}H-NMR of PEG-OH (polymer concentration: 10 mg/mL, solvent: Toluene-d8, and temperature: 80 °C).

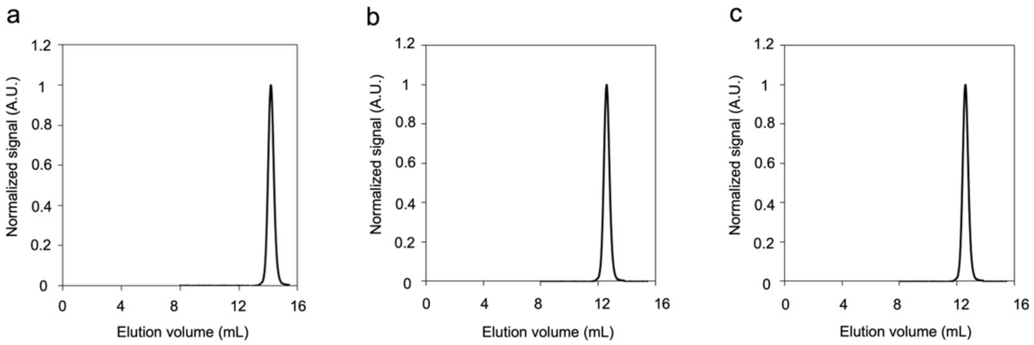

Supplementary Figure 3. SEC curves of (a) PEG-OH, (b) PEG-PECH, and (c) PEG-PG (Polymer concentration: 1 mg/mL, solvent: DMF with 10 mM lithium chloride, temperature: 40 °C).

Supporting Table 1. Elementary analysis of PEG-PECH

Polymer	Cl (%)	C (%)	H (%)	O (%)
PEG-PECH	14.7	48.6	7.67	29.0

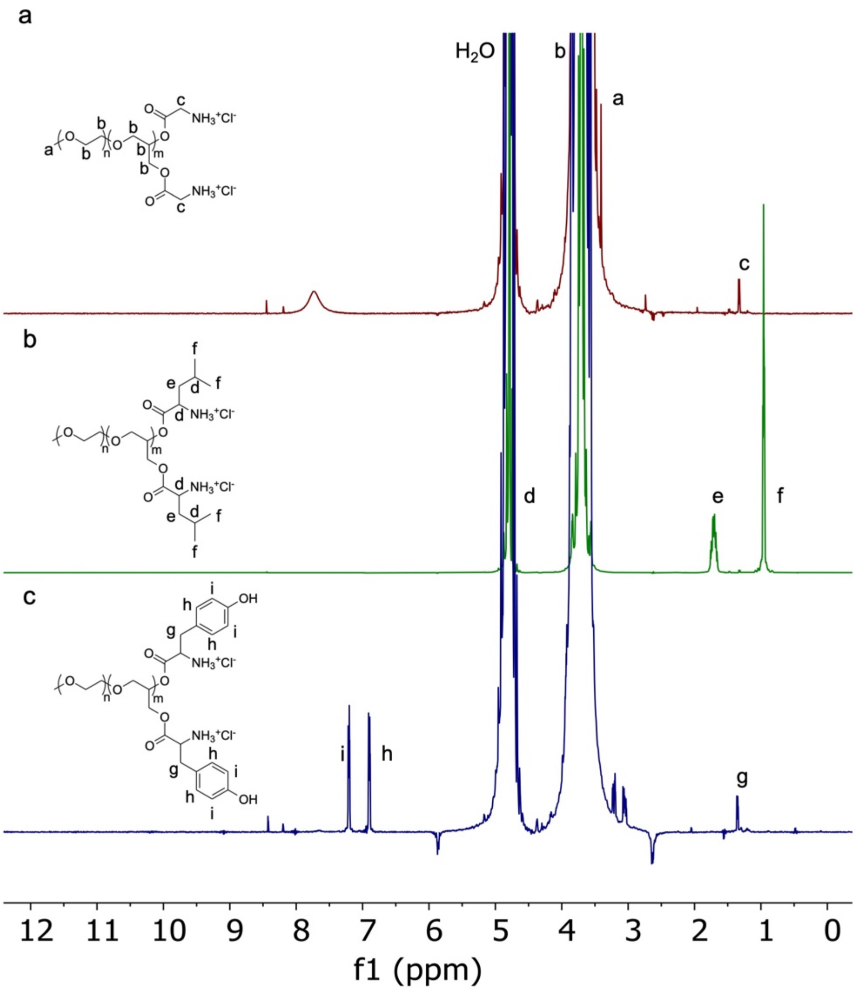

Supplementary Figure 4. ^{1}H-NMR of a) PEG-PGGly, b) PEG-PGLeu and c) PEG-PGTyr (polymer concentration: 10 mg/mL, solvent: D_2O, and temperature: 25 °C).

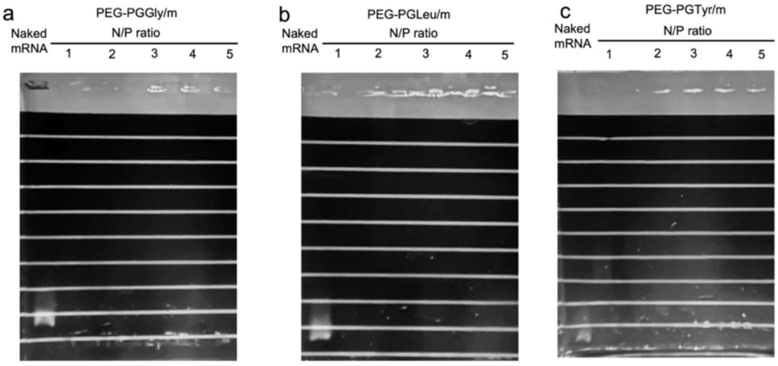

Supplementary Figure 5. Electrophoretic analysis of the PEG-PGGly/m (a), PEG-PGLeu/m (b) and PEG-PGTyr/m (c) at N/P ratios ranging from 1 to 5 on 1% agarose gel (15 µL sample solution containing 500 ng of mRNA were applied to each well and mRNA was visualized using Midori Green Direct dye).

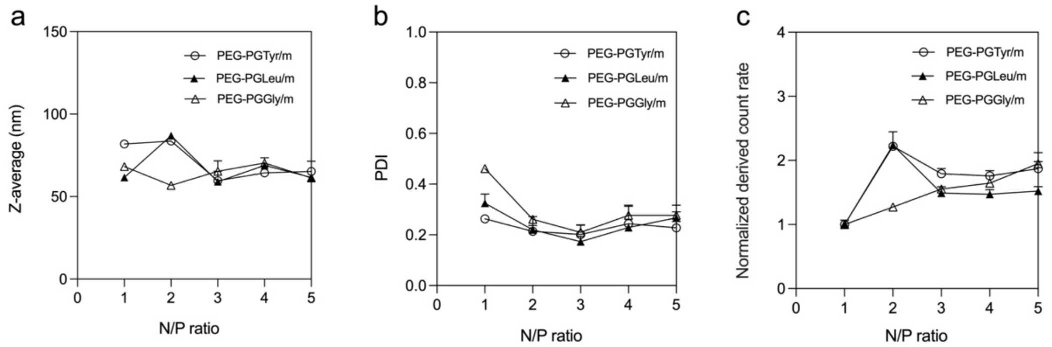

Supplementary Figure 6. DLS characterization of micelles from PEG-PGGly, PEG-PGleu and PEG-PGTyr. (a) Z-average diameter, (b) polydispersity index (PDI), and (c) normalized derived count rate (normalization with derived count rate of N/P = 1) of micelles in 10 mM HEPES buffer.
